# Commentary: Association between wine consumption and cancer: a systematic review and meta-analysis

**DOI:** 10.3389/fnut.2024.1511706

**Published:** 2024-12-13

**Authors:** Maribel Lucerón Lucas-Torres, Ivan Cavero-Redondo, Vicente Martinez-Vizcaino, Bruno Bizzozero-Peroni, Carlos Pascual-Morena, Celia Alvarez-Bueno

**Affiliations:** ^1^Health and Social Research Center, Universidad de Castilla-La Mancha, Cuenca, Spain; ^2^Faculty of Health Sciences, Autonomous University of Chile, Santiago, Chile; ^3^Instituto Superior de Educación Física, Universidad de la República, Montevideo, Uruguay

**Keywords:** wine, wine consumption, adults people, alcohol consumption, cancer

As suggested in the comment by Natella et al., the sentence in the abstract could be removed or reworded, as the meta-analysis does not support the idea that wine consumption offers protection against several types of cancer and may be confusing for the reader. This error has occurred because the abstract is very generic without specifying the association between wine and cancers one by one owing to the limited number of words allowed, but as mentioned in the commentary, in the full article, this problem does not exist. Although the pooled estimations for meta-analyses show no association, which is what the authors intended to convey, the lower ranges of the confidence intervals reflect an association that may be clinically useful, even if not statistically significant. Certainly, the current wording could lead to confusion in the interpretation of these results.

The comment authors pointed out a trend with the omission of meta-analyses for certain types of cancer, and they encourage the authors to clearly specify the types of cancer for which a meta-analysis was carried out and those for which it was not possible. The main reason for this limitation is the limited number of articles available for some cancers in particular. Despite this limitation, a graphical representation has been included for these specific cases, providing the reader with a visual summary of the risk of wine consumption with the available studies, even when a meta-analysis could not be performed. The intention of this graphical representation is to show where the trend in wine consumption and certain types of cancer is pointing so that future studies can support this trend. Substantial heterogeneity and publication bias were investigated and included in the study. In addition, limitations were included regarding the heterogeneity of the included studies in reporting alcohol exposure, as they differed in the methods used to measure wine consumption and did not report the specific volume of wine consumed. This lack of data has influenced the quality of our results because the associations between wine consumption and the development of different types of cancer could not be analyzed by type of wine, amount of wine consumed or sex.

Wine, in addition to ethanol, contains nutrients, micronutrients, proteins, and a large amount of phenolic substances that affect human health (1). The most beneficial components of wine are polyphenols, which are found in the solid parts of the grape, i.e., the skins, seeds, and stems (2). There are other components that are not polyphenols but also have positive effects on human health, such as melatonin or gallic acid (3, 4). Polyphenols have antioxidant, anti-inflammatory, anticarcinogenic, and antiaging effects (5), inhibit platelet aggregation, have vasorelaxant effects, modulate lipid metabolism, have neuroprotective effects (6), regulate the microbiota (7, 8), and even have chemopreventive effects, among many other benefits (9). Polyphenols include resveratrol, the polyphenol that has been analyzed the most thus far (10, 11). As a good polyphenol, resveratrol offers multiple benefits to the body by inhibiting LDL oxidation and suppressing platelet aggregation, has antiatherosclerotic properties, promotes vascular relaxation, and has endothelial protective functions. In addition, it regulates various substances, such as nitric oxide synthase, thioredoxin-1, hemeoxygenase-1, vascular endothelial growth factor, manganese superoxide dismutase, and caveolin-1, thus contributing to the prevention of oxidative stress (12, 13, 22). However, the role of resveratrol and flavonoids in health, as the commentary says, is still under study for several reasons, the first of which is from a pharmacological point of view: there is an interaction between resveratrol and certain drugs, such as oral anticoagulants, causing the drug to reach the blood in lower quantities (14, 15). On the other hand, at low doses, resveratrol acts as a cardioprotective agent, but at high doses, it induces apoptosis in cancer cells by exerting a death signal, depressing cardiac function, and increasing the risk of myocardial infarction (16). With respect to resveratrol in wine, previous evidence shows that the resveratrol dose in wine is at average levels of 7 mg L-1 in red wine and 0.5 mg L-1 in white wine (17); although resveratrol in the human body is well-absorbed, its free fraction in the blood is low and therefore has a low bioavailability compared with that reported in *in vitro* studies (18). In addition, evidence over the years has revealed multiple benefits of resveratrol in wine, as it is one of the most interesting polyphenols in wine, as it provides benefits for a wide range of medical problems by reducing oxidative stress and exerting anti-inflammatory and antimutagenic effects on diseases such as cancer (9).

On the other hand, although the article focuses on wine consumption, as is reflected in the wording of the article, and some data may indicate that wine consumption may have protective or beneficial effects on health, the comment authors believe it is important to highlight that it is still a type of alcohol and that, as such, it is necessary to draw attention to moderate consumption, but it is true that this work does not show any association, neither negative nor positive, so neither can report that wine consumption increases the risk of cancer. Notably, alcohol consumption is a risk factor for the development of this pathology, which, together with tobacco, may have a concomitant effect (19). Although the evidence is controversial and seems that the type of alcohol may also influence the development of this pathology (20), the authors want to stress that although the evidence reports that light-moderate consumption of wine may bring health benefits, caution should be exercised with its consumption, especially in at-risk populations. Therefore, we may highlight that we do not want to recommend wine consumption but rather synthesize the existing evidence on wine consumption, always remembering that safe consumption is zero, that inadequate consumption can be very harmful to health, for many pathologies and that a lifelong abstainer starting to consume can have very detrimental effects on one's health (21).

Finally, in addressing the last point about retracting this scientific article, the authors consider that it is not appropriate because, with our article, we have synthesized data from original studies on wine consumption and different types of cancer; the systematic review and meta-analysis have been carried out in a methodologically correct way, so we believe that it does not justify the article being retracted.
